# Modality-based attention and dual-stream multiple instance convolutional neural network for predicting microvascular invasion of hepatocellular carcinoma

**DOI:** 10.3389/fonc.2023.1195110

**Published:** 2023-06-26

**Authors:** Zhi Li, Yutao Wang, Yuzhao Zhu, Jiafeng Xu, Jinzhu Wei, Jiang Xie, Jian Zhang

**Affiliations:** ^1^ School of Medicine, Shanghai University, Shanghai, China; ^2^ Shanghai Universal Medical Imaging Diagnostic Center, Shanghai University, Shanghai, China; ^3^ The First Affiliated Hospital of Ningbo University, Ningbo, China; ^4^ School of Computer Engineering and Science, Shanghai University, Shanghai, China

**Keywords:** hepatocellular carcinoma, microvascular invasion, multiple instance learning, attention mechanism, MRI

## Abstract

**Background and purpose:**

The presence of microvascular invasion (MVI) is a crucial indicator of postoperative recurrence in patients with hepatocellular carcinoma (HCC). Detecting MVI before surgery can improve personalized surgical planning and enhance patient survival. However, existing automatic diagnosis methods for MVI have certain limitations. Some methods only analyze information from a single slice and overlook the context of the entire lesion, while others require high computational resources to process the entire tumor with a three-dimension (3D) convolutional neural network (CNN), which could be challenging to train. To address these limitations, this paper proposes a modality-based attention and dual-stream multiple instance learning(MIL) CNN.

**Materials and methods:**

In this retrospective study, 283 patients with histologically confirmed HCC who underwent surgical resection between April 2017 and September 2019 were included. Five magnetic resonance (MR) modalities including T2-weighted, arterial phase, venous phase, delay phase and apparent diffusion coefficient images were used in image acquisition of each patient. Firstly, Each two-dimension (2D) slice of HCC magnetic resonance image (MRI) was converted into an instance embedding. Secondly, modality attention module was designed to emulates the decision-making process of doctors and helped the model to focus on the important MRI sequences. Thirdly, instance embeddings of 3D scans were aggregated into a bag embedding by a dual-stream MIL aggregator, in which the critical slices were given greater consideration. The dataset was split into a training set and a testing set in a 4:1 ratio, and model performance was evaluated using five-fold cross-validation.

**Results:**

Using the proposed method, the prediction of MVI achieved an accuracy of 76.43% and an AUC of 74.22%, significantly surpassing the performance of the baseline methods.

**Conclusion:**

Our modality-based attention and dual-stream MIL CNN can achieve outstanding results for MVI prediction.

## Introduction

Hepatocellular carcinoma (HCC) is a prevalent cancer that ranks as the third leading cause of cancer-related death (1). Even with treatment such as hepatic resection surgery or liver transplantation, patients still face a high risk of recurrence. Microvascular invasion (MVI) has emerged as a critical prognostic factor for HCC, with patients having MVI exhibiting earlier recurrence than those without it (2, 3). Early identification of MVI before surgery is pivotal in determining suitable treatment strategies and preoperative adjuvant therapy (4). Nonetheless, traditional identification methods rely on postoperative pathological examination, thus making preoperative assessment a challenge. Hence, there is an urgent need to develop new methods for preoperatively assessing MVI.

Numerous prior studies have investigated the application of radiomics in predicting MVI in patients with HCC. In a two-center study, Tian et al. (5) utilized gadolinium ethoxybenzyl diethylenetriamine pentacetic acid-enhanced MRI to assess MVI in small, solitary HCC (<= 3 cm). Matteo et al. (6) identified radiomic MVI predictors in nodules by analyzing the zone of transition from contrast-enhanced computed tomography, while Jiang et al. (7) explored the contribution of F-18-fluorodeoxyglucose positron emission tomography/computed tomography (F-18-FDG PET/CT) radiomic features in HCC and intrahepatic cholangiocarcinoma (ICC) classification and MVI prediction prior to surgery. These studies have all demonstrated promising performance. Despite their favorable outcomes, radiomics methods have several limitations. Precise segmentation of lesions and the selection of hand-crafted features, which rely on expert knowledge and require significant annotation time, are some examples.

In recent years, deep learning-based methods have gained popularity for analyzing medical images, including tasks such as lesion classification (8), segmentation (9), and detection (10). These methods have the advantage of extracting informative features with minimal preprocessing, allowing for autonomous end-to-end predictions (11). Several studies have explored the use of deep learning for predicting MVI in HCC patients. Gao et al. (12) proposed an ensemble learning algorithm based on the lesion patch of HCC non-contrast T1 weighted MRI. Zeng et al. (13) developed an attention-based deep learning model for MVI prediction using the intra-voxel incoherent motion model of diffusion-weighted MRI. Liu et al. (14) used a deep learning model with a combination of CT images of arterial phase and patients’ clinical factors to assess MVI status. However, these methods only take into account a single slice or patch of medical images, thereby overlooking the overall contextual information of the lesion. To address this limitation, some studies have focused on incorporating partial or entire lesion portions as input with 3D CNN. Zhou et al. (15) demonstrated the effectiveness of a deeply supervised CNN with multiple stages of contrast-enhanced MRI 3D blocks for MVI prediction. Zhang et al. (16) built a fusion 3D model combining three MR sequences for the noninvasive prediction of MVI in HCC. Jiang et al. (17) developed a Radiomics-Radiological-Clinical Model and 3D-CNN models based on three CT phases of the volume data to generate the MVI assessment. Nevertheless, these methods come with drawbacks such as high GPU memory consumption and difficulty in training due to their large number of parameters and high complexity (18). As a result, these methods are often designed with shallow 3D network layers, which may limit their ability to extract informative features.

To overcome the limitations of prior research, we introduce a novel approach called the modality-based attention and dual-stream MIL CNN. Our method addresses the issue of overlooking vital contextual information by leveraging all the slices from 3D lesions. What’s more, unlike 3D CNNs that can be computationally demanding and difficult to train by directly inputting the entire tumor, our approach uses a 2D feature extractor to initially process each lesion slice and then aggregates these features to represent the entire lesion. This strategy balances computational efficiency with adequate lesion information extraction. Additionally, our model includes a modality attention module to help it focus on important MRI sequences, and a dual-stream MIL aggregator to prioritize critical slices. These modules emulate the decision-making process of doctors, making our model more explainable.

## Methods

### Study participants

This retrospective study was approved by the Ethics Committee of the Local Imaging Diagnostic Center. The study aimed to analyze the MRI characteristics of HCC patients who underwent surgery between April 2017 and September 2019 for MVI prediction. A total of 415 consecutive patients diagnosed with HCC by pathological results were enrolled. Patients with/without MVI was 146 and 137. The T2-weighted, arterial phase, venous phase, delay phase, and apparent diffusion coefficient MRI were collected from each eligible patient for further analysis.

**Figure 1:**
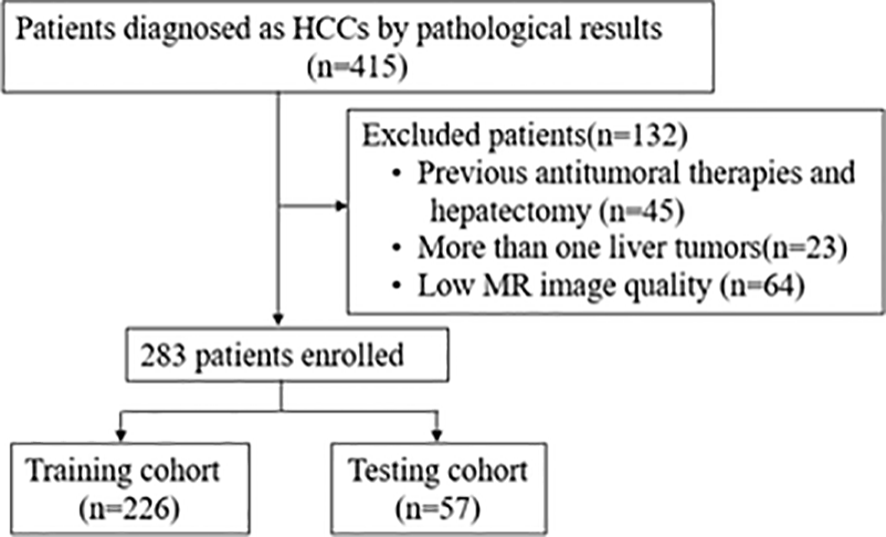
Flowchart of patient recruitment. HCC, Hepatocellular carcinoma; MR, Magnetic Resonance.

To be eligible for the study, patients had to meet certain criteria, including a histopathologically diagnosed HCC with confirmed MVI status and an effective dynamic-contrast enhanced MRI available within one month before surgery. Patients who had undergone antitumoral therapies or hepatectomy, had more than one liver tumor, or had low MRI quality that could affect the delineation of the region of interest (ROI) were excluded from the study. Finally, a total of 283 patients were included and the recruitment process is as shown in **Figure 1**.The clinical information of each patient including age, sex, ratio of maximum to minimum tumor diameter (Max/Min-TD), and tumor location were collected by clinicians with more than 5 years of clinical experience. Clinical information was shown in **Table 1**. The requirement for written informed consent was waived by the institutional review board.

**Table 1:** Clinical characteristics in the MVI positive and MVI negative groups.

Variables	MVI+ (N= 146)	MVI- (N= 137)	P Value
Sex, no.(%)			0.968
Male	122(83.56)	118(86.13)	
Female	24(16.44)	19(13.87)	
Age (years), mean ± SD	53.12 ± 12.40	51.54 ± 10.18	0.486
Max/Min-TD, mean ± SD	3.21 ± 0.87	1.83 ± 0.52	0.003
Tumor location, no. (%)			0.146
Right lobe of liver	125(85.61)	116(84.67)	.874
Left lobe of liver	21(14.39)	17(15.33)	

MV represent MVI positive; MVI, represent MVI negative; Max/Min-TD, the ratio of maximum to minimum tumor diameter.

### Data preprocessing

**Figure 2:**
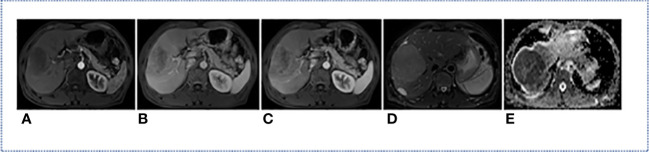
MRI modality of one HCC Patient. **(A)** Arterial Phase; **(B)** Delay Phase; **(C)** Venous Phase; **(D)** T2-Weighted; **(E)** apparent diffusion coefficient.

**Figure 3:**
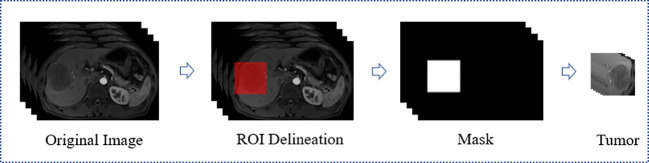
Flowchart of preprocess for MRI. ROI, Region of interest.

All MRI used in this study were acquired from Siemens medical image workstation (syngo.via) for post-processing. The contrast-enhanced MRI consisted of images of T2-weighted, arterial phase, venous phase, delay phase, and apparent diffusion coefficient (**Figure 2**). Prior to the training process, four preprocessing steps were performed (**Figure 3**).

Firstly, A radiologist with five years of experience utilized the ITK-SNAP software (https://www.radiantviewer.com) to delineate the ROI and generated a mask for each layer of the arterial phase MRI. The delineation was then reviewed and confirmed by a radiologist with 20 years of experience. Each ROI was a bounding box that approximately 10mm larger than the tumor boundary and centered on the tumor. This delineation method not only covered the tumor boundary which would helpful to MVI prediction, but also saved a considerable amount of annotation time compared to fine labeling along the tumor edge. Secondly, to integrate information from all modalities, the delay phase, venous phase, apparent diffusion coefficient and T2-weighted MRI were aligned with the arterial phase through image registration. Thirdly, the five MRI modality were tailored according to the mask to obtain the 3D lesion area. Fourthly, the pixel value of each slice was normalized using Z-score standardization, and each slice was uniformly scaled to 64×64 pixels before input to the network. During the training stage, data augmentation was performed by applying a random horizontal rotation and flip with a probability of 0.5. Overall, the preprocessing steps were aimed at improving the quality of the input data and augmenting the dataset to enhance the performance of the neural network used in this study.

### Multiple instance learning

The process of diagnosing diseases by doctors can be effectively simulated through the use of MIL, which is a type of weakly supervised learning approach (19). In this approach, the unit of training and testing is a “bag” composed of multiple instances, with each instance having an uncertain label (20). The label of a bag is usually known, and it is considered positive if it contains at least one positive instance, and negative otherwise. This method mirrors the diagnostic process used by doctors, who carefully examine all slices of a 3D medical image. If any abnormalities are detected in a single slice, the patient can be diagnosed with a problem.

In the field of computer-aided diagnosis, the MIL framework is widely used in histopathologic whole-slide images (21) and (22). For binary classification, given a bag B = {(*x_1_, y_1_*),…, (*x_n_, y_n_*)}, where *x_i_* is an instance and *y_i_* is the corresponding instance label, with *y_i_* ∈{0, 1}, the relationship between the bag labels and the instance labels can be expressed by the following formula.


$$c(B)=\left\{\begin{array}{c} 0,\text{ iff}\sum y_{i}=0\\ 1,\text{ otherwise} \end{array}\right\}$$

### Proposed framework

Our model is inspired by the process of disease diagnosis performed by doctors. During this process, doctors examine all MRI slices, and if at least one slice shows abnormalities, they determine the presence of a disease; otherwise, they conclude that there is no disease. This process bears a resemblance to the concept of MIL, which is why we adopted a MIL approach in our model. Additionally, when dealing with multi-modal MRI, doctors focus on certain important MRI modalities and, finally, pay attention to specific crucial MRI slices. These aspects also serve as inspiration for our model construction.

**Figure 4:**
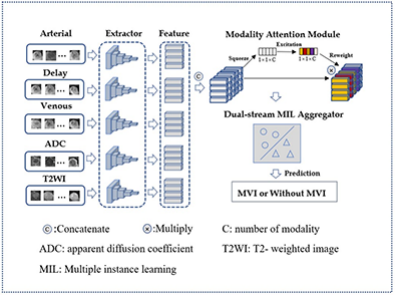
The framework of the proposed method.

Our proposed architecture of this paper was shown in **Figure 4** and can be divided into three main parts: a feature extractor, a modality attention module, and a dual-stream instances feature aggregation module. The feature extractor is responsible for extracting instance features, while the modality attention module emulates the decision-making process of doctors and helps the model to focus on the most important MRI sequences. The dual-stream instances feature aggregation module aggregates the features of all instances into bag features, allowing the model to concentrate on the critical MRI slices.

In this study, the MRI of a patient’s tumor is treated as a bag, with each slice of the lesion considered an instance. After preprocessing, the five MRI modalities of one patient, each with a dimension of s×64×64, are simultaneously input into the network. Here, s represents the number of slices in the MRI volume, and 64 represents the width and height of the slice. Each MRI modality is first processed by a feature sharing extractor, which produces a feature tensor with a dimension of s×128. After concatenating the feature tensors of the five MRI modalities, the dimension of the instance feature tensor is still 1×128. The tensor is then fed into the modality attention module, which assigns higher weights to the MRI modality with the highest predictive effect. The features of the instances are then aggregated to form the bag features, which are used to produce the final prediction results.

### Feature extractor

**Figure 5:**
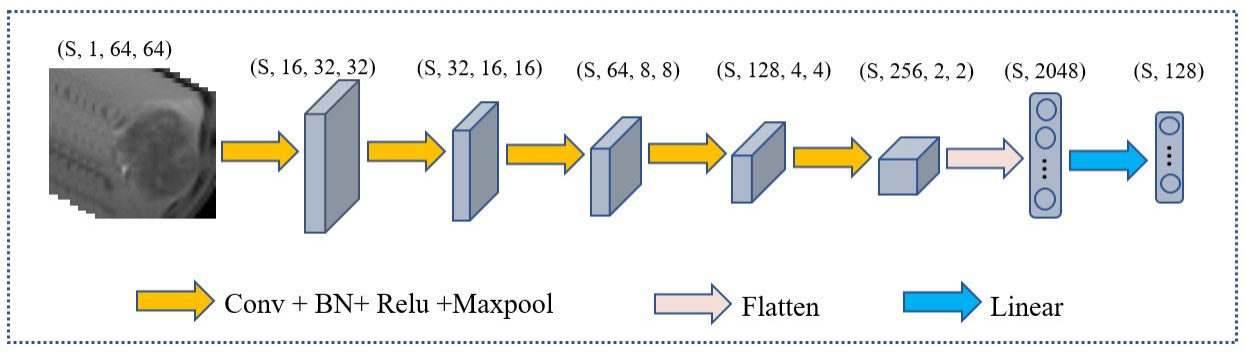
The architecture of the extractor. (s, 1, 64, 64) represent the number, channels, length, and width of slice in MRI respectively.

**Figure 5** illustrates the architecture of the feature extractor, which comprises five convolutional layers and one fully connected layer. Each convolutional layer is accompanied by a rectified linear unit activation function, a batch normalization layer, and a pooling layer. The purpose of the batch normalization layer is to expedite model convergence, the relu layer enhances the model’s nonlinearity, and the pooling layer decreases the size of the feature map. Specifically, every convolutional layer has a convolutional kernel size of 3×3 and the maxpool is 2×2. The number of channels in each convolutional layer is 16, 32, 64, 128, 256. The neurons of fully connected layer is 128.

In the feature extractor, the input is a 1×64×64 MRI slice, and after five convolutional layers, the output feature dimension is 256×2×2. Upon flattening, the feature map’s dimension from the convolutional layers is 4096, which is reduced to 128 by a fully connected layer.

### Modality attention module

Doctors usually rely on multiple MRI modalities to make diagnostic decisions. In order to replicate this process, a modality attention module was introduced, as shown in **Figure 4**, building upon previous research (23) and senet model (24). This module replaces the channels in senet with the MRI modality number used in this study, allowing for automatic perception of the importance of different MRI modalities. The module consists of three main operations: squeeze, excitation, and reweight.

Here, Let M = {*m_1_,m_2_,m_3,_ m_4_,m_5_*} refers to the features of five MRI modalities. *m_k_* ϵℝ ^S×128^ (k = 1, 2, 3, 4, 5) denote the modal feature tensor of the kth modality, where s represents the number of slices and 128 represents the feature tensor of each slice. a global average pooling was used to squeeze a modality-wise descriptor, as follows:


$${\text{d}}_{\text{k}}=\frac{1}{\text{s}\times 128}\sum_{i=1}^{s}\sum_{j=1}^{128}m_{k}\left(i,j\right)$$

where *m*_k_(*i,j*) represents the *j*th element in the *i*th slice of m*_k._* The excitation operator maps the descriptor to a set of modality weights. it was simulated SENet by two fully connected layers and a relu structure. It can be described as follow: $a=W_{2}relu\left(W_{1}d\right)$ where *d* = {*d_1_,d_2_,d_3,_d_4_,d_5_*} is the modality-wise descriptor vector, *W_1_* and *W_2_* are the weight vector of the two fully connected layers, and *a* = {*a_1_,a_2_,a_3_*,*a_4_,a_5_*} is the attention vector.

The reweight operator ensures that specific MRI modalities are emphasized by multiplying the attention vector *a* = {*a_1_,a_2_,a_3_*} with the original multi-modality features M = {*m_1_,m_2_,m_3,_ m_4_,m_5_*}. It can be described as follow:


$$\tilde{m}=\sum_{i=1}^{5}a_{i}m_{i}$$

Where $\tilde{m}$ represents the features of five MRI modalities after reweighting.

### Dual-stream instance multiple instance aggregator

**Figure 6:**
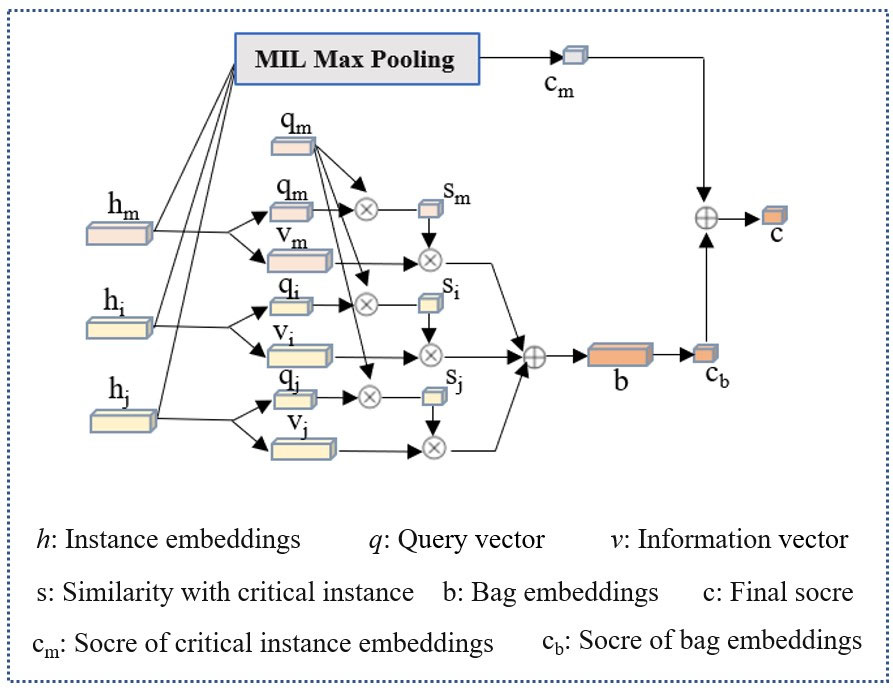
Overview of the dual-stream MIL aggregator.


Apart from the imaging modality, doctors also pay close attention to the key MRI slices, as these slices provide a clearer view of lesion information. To replicate this process, we incorporated the dual-stream MIL aggregator module (25) into our framework, which was initially designed to operate on pathological image patches (as illustrated in the **Figure 6**). Let *B* = {*x_1_, …, x_n_*} represent a bag of MRI slices of a particular modality, where *x_i_* represent the instance *i.* After *x_i_* passing through the feature extractor module and modality attention module, it can be projected onto an embedding of *h_i_* ∈ ℝ ^128×1^. The first stream of the module employs an instance classifier on each instance embedding and performs MIL max-pooling on the scores to obtain the critical instance *h_m_* and the highest score *c_m_*:

(1) $$c_{m}(B)=\max\{W_{0}h_{0},\ldots ,W_{0}h_{n-1}\}$$

where *W_0_* is a weight vector of fully connected layer and represents a classifier. *W_0_h_i_* means get the prediction scores of *h_i_* by classifier. Then the max{*W_0_h_0_,…,W_0_h_n-1_*} means get the max score in *W_0_h_i_*.

The second stream combines all instance embeddings to generate a bag embedding, which is scored by a bag classifier. Specifically, each instance embedding *h_i_* (including *h_m_*) were converted into two vectors, query *q_i_* ∈ ℝ ^128×1^ and information *v_i_* ∈ ℝ ^128×1^ by:


(2) $$q_{i}=W_{q}h_{i},\;v_{i}=W_{v}h_{i}\cdot \cdot i=0,\ldots ,n-1$$

where *W_q_* and *W_v_* is a weight matrix, query *q_i_* and query *v_i_* are another information representation of *h_i_* by linear operation of *W_q_h_i_ and W_v_h_i_*. Then a similarity *U*(*h_i_*, *h_m_*) between an instance to the critical instance was defined by


(3) $$U(h_{i},h_{m})=\frac{\exp(\langle q_{i},q_{m}\rangle )}{\sum_{k=0}^{N-1}\exp(\langle q_{k},q_{m}\rangle )}$$

$q_{i},q_{m}\;$ represents the inner product of any vectors *q_i_* and critical instance vectors *q_m_*, also it represent the cosine similarity of *q_i_* and critical instance vectors *q_m_*. Then a softmax layer to calibrate the weights of all *U*(*h_i_*, *h_m_*) to ensure that their sum was 1. The bag embedding, denoted by b, is obtained by performing a weighted element-wise sum of all instances’ information vectors *v_i_*, where the weights are determined by their similarity to the critical instance.


(4) $$b=\sum_{i}^{N-1}U(h_{i},h_{m})v_{i}$$

The bag score *c_b_* is then determined by:


(5) $$c_{b}(B)=W_{b}b$$

where *W_b_* is a weight vector for binary classification. *W_b_b*means get the prediction scores of *b* by classifier *W_b_*. The final bag score is the average of the two streams’ scores:


(6) $$c(B)=\frac{1}{2}(c_{\text{m}}(B)+c_{\text{b}}(B))$$

Where *c_m_* (*B*) represent the max instance score, *c_b_* (*B*) represent the bag score, and *c_m_* (*B*) represent final bag score for prediction.

### The implementation

We utilized the Pytorch open-source deep learning framework to implement the proposed framework. All experiments were conducted on a Dell T640 tower server deep learning workstation, which had two independent NVIDIA GeForce RTX 2080Ti graphics cards and two Intel Xeon Silver 4110 CPUs. For the experiments, we employed the cross-entropy loss function and the Adam optimizer. The gradient descent method used an initial learning rate of e-4, with a decay rate of 5e-3. The batch size of each iteration was set to 1, and the number of training epochs was 100.

### Statistical analysis

In this study, the dataset was divided into a training set and a testing set in a ratio of 4:1. Five-fold cross-validation was employed to evaluate model performance, enhancing stability and reducing evaluation bias. Categorical variables were statistically analyzed using the chi-square test and continuous variables were analyzed using the student’s t test to determine significant differences between the MVI and non-MVI groups. To assess the performance of the models, five performance metrics: accuracy, sensitivity, specificity, positive predictive value (PPV), negative predictive value (NPV) and area under the curve (AUC) were computed. The 95% confidence intervals (CIs) for AUC was estimated using the DeLong method. The performance of different models were compared using the student’s t-test, and a P-value of less than 0.05 was considered as indicative of significant difference. Statistical computing was implemented with the Scipy package, an open-source data processing tool based on the Python.

## Result

### Patient characteristics

The clinical characteristics of the cases are presented in **Table 1**. Among the 283 cases, 146 (53.4%) were positive for MVI, while 136 (46.6%) were negative for MVI. There were no significant differences in terms of sex, age, and tumor location between the MVI positive and MVI negative groups. However, the ratio of maximum to minimum tumor diameter (Max/Min-TD) was significantly higher in the MVI positive group (3.21 ± 0.87) compared to the MVI negative group (1.83 ± 0.52) with a P-value of 0.003.

### Performance analysis of different module included

The ablation experiments were conducted to demonstrate the effectiveness of our method. The benchmark were obtained by removing the framework’s modality attention and dual-stream instance aggregator module. Then, these two modules were added to the benchmark, either separately or together. The results were displayed in **Table 2**.

**Table 2:** Model performance of different modules included in the proposed framework.

Benchmark	Attention	DS Aggregator	Accuracy (%)	Sensitivity (%)	Specificity (%)	NPV (%)	PPV (%)	AUC
✓			67.14	**73.89**	60.30	67.38	67.77	0.6710
✓	✓		69.64	72.22	65.93	70.45	69.80	0.7023
✓		✓	75.71	69.29	**81.18**	72.93	**83.09**	0.7336
✓	✓	✓	**76.43**	71.41	80.36	**74.16**	81.74	**0.7422**

Values in bold black font represent the best performance in each column.

The benchmark achieved an accuracy of 67.14% and an AUC of 0.6710. Upon adding the modality attention module, the accuracy and AUC improved to 69.64% and 0.7023, respectively. Similarly, adding the dual-stream instance aggregation module resulted in an accuracy and AUC of 75.71% and 0.7336. Combining both modules yielded the best prediction performance, with an accuracy of 76.43% and an AUC of 0.7422. Notably, this represents a 13.84% and 10.61% improvement in accuracy and AUC compared to the benchmark. This ablation experiments confirmed the importance of the modality attention and dual-stream instance aggregator modules in improving prediction performance. Specifically, combining both modules yielded the greatest improvement in accuracy and AUC, demonstrating the efficacy of our proposed method.

### Performance with different convolutional layers of feature extractor

In order to extract features from 2D slices within the bag, we designed a simple CNN as a feature extractor. However, the number of layers within the CNN can significantly impact the model’s performance. Therefore, we conducted experiments to determine the optimal number of layers to use. The performance of the CNN was evaluated with varying numbers of convolution layers, as shown in **Table 3**. Specifically, we tested the CNN with 3, 4, 5, 6 and 7 layers, which resulted in AUC values of 0.7093, 0.7339, 0.7422, 0.7417 and 0.7317, respectively. Overall, setting the number of layers to 5 yielded the best results. When the number of layers was less than or greater than 5, the performance deteriorated. Therefore, it is advisable to use 5 layers for optimal results.

**Table 3:** Model performance of different convolutional layers for Feature Extractor.

Conv-Layers	Accuracy (%)	Sensitivity (%)	Specificity (%)	NPV (%)	PPV (%)	AUC
3	71.79	73.16	69.79	73.75	72.60	0.7093
4	73.93	**78.46**	69.07	74.91	73.31	0.7339
5	**76.43**	71.41	**80.36**	74.16	**81.74**	**0.7422**
6	75.00	72.92	77.22	73.80	77.73	0.7417
7	74.29	77.77	70.72	**76.97**	74.38	0.7413

Values in bold black font represent the best performance in each column.

### Performance with individual MRI modality

Different modalities of MRI can reveal distinct characteristics of tumors (26), and physicians tend to focus on specific modalities to make informed decisions. In order to assess the correlation between the model’s performance and realistic clinical diagnosis, we evaluated the model’s results under individual MRI modalities. As shown in **Table 4**, the arterial phase MRI was found to be the most effective modality for predicting MVI, with accuracy, sensitivity, NPV, and AUC values of 72.50%, 76.73%, 76.19%, and 0.6974, respectively. The venous phase MRI was the second most effective modality, followed by the ADC, Delay MRI, and T2 MRI in descending order of effectiveness. The best results were obtained when all MRI modalities were added with accuracy, specificity, PPV, and AUC values of 76.43%, 80.36%, 81.74% and 0.7422.

**Table 4:** Model performance under individual MRI modality.

Modality	Accuracy (%)	Sensitivity (%)	Specificity (%)	NPV (%)	PPV (%)	AUC
**Arterial**	72.50	**76.73**	68.81	**76.19**	72.30	0.6974
**Delay**	68.57	68.15	69.38	69.16	72.99	0.6303
**Venous**	70.00	75.80	63.52	70.69	70.44	0.6813
**ADC**	70.00	60.86	77.98	66.65	**75.35**	0.6323
**T2**	64.64	65.82	62.56	64.78	66.65	0.5756
**combined**	**76.43**	71.41	**80.36**	74.16	**81.74**	**0.7422**

Values in bold black font represent the best performance in each column.

### Performance of data augmentation

In this study, a data augmentation strategy was employed, including random horizontal and vertical flips within 90°, due to the limited size of the dataset. To assess the impact of this strategy, the performance of the model was compared with and without data augmentation. The results, presented in the **Table 5**, indicate that the model performs significantly better when data augmentation is utilized. This improvement may be attributed to the fact that the combination of random data augmentation for each instance greatly increases the number of bags, leading to a more diverse and robust dataset.

**Table 5:** Model performance with data augmentation.

Data augmentation	Accuracy (%)	Sensitivity (%)	Specificity (%)	NPV (%)	PPV (%)	AUC (%)
No	70.71	70.26	70.22	69.75	72.30	0.6804
Yes	**76.43**	**71.41**	**80.36**	**74.16**	**81.74**	**0.7422**

Values in bold black font represent the best performance in each column.

### Comparison with other methods

**Figure 7:**
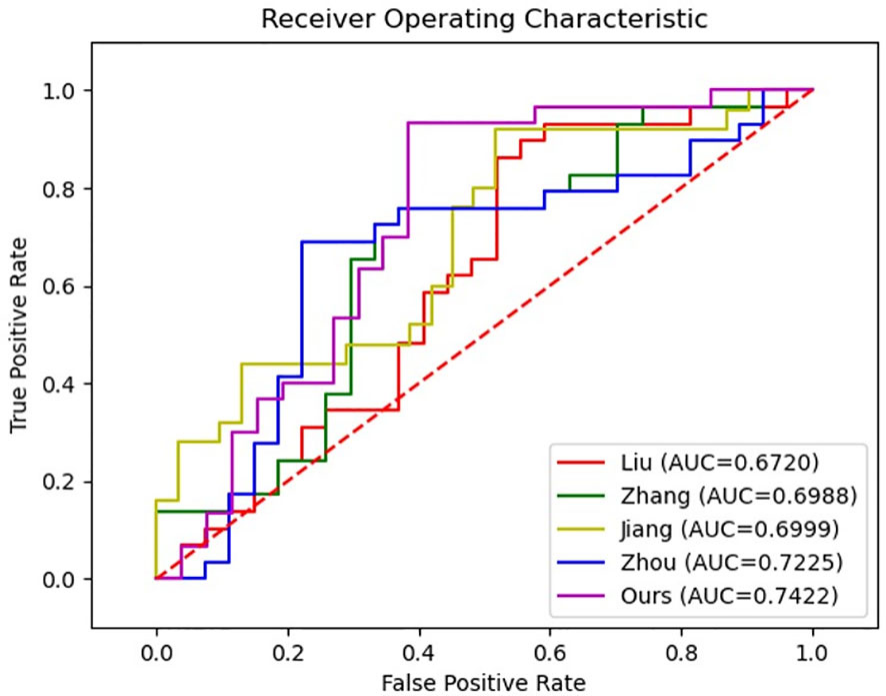
ROC curves of our method and other methods.

In order to further validate the effectiveness of our proposed method for diagnosing MVI, we conducted a comparative analysis with four representative MVI diagnostic approaches based on deep neural networks. However, since each study utilized different datasets, directly comparing the results would have been unfair. Therefore, we reproduced these methods on our datasets to ensure a fair comparison. Specifically, in reproducing Liu et al. (14), we employed a five-branch network of ResNet18 (27) to extract features from the largest slice of each MRI modality, including T2-weighted, arterial phase, venous phase, delay phase, and apparent diffusion coefficient. The output features of each network were then combined for the final prediction. Similarly, models following Zhou et al. (15), Jiang et al. (17), and Zhang et al. (16) with five-branch network were constructed. In reproducing Zhou’s approach, the entire HCC tumor was inputted instead of using 3D block lesions. Additionally, deep supervision was included for each network by employing the cross-entropy loss function. The results of the comparative analysis are presented in **Table 6** and **Figure 7**, and it is clearly displayed that the proposed method outperformed the others in terms of accuracy, specificity, PPV, and AUC. Although there was no significant difference in the values of AUC, superior performance across multiple metrics was achieved by the proposed approach.

**Table 6:** Comparison with other methods.

Methods	Accuracy (%)	Sensitivity (%)	Specificity (%)	NPV (%)	PPV (%)	AUC(95% CI)	*P* value
Liu (14)	66.79	73.05	61.35	67.88	**67.65**	0.6720 (0.5884, 0.7629)	0.0572
Zhang (16)	70.00	70.87	68.89	69.52	**70.90**	0.6988 (0.6624, 0.7384)	0.1207
Jiang (17)	70.36	76.29	63.69	72.13	**70.32**	0.6999 (0.6272, 0.7785)	0.1065
Zhou (15)	72.50	**82.14**	62.35	**76.92**	**70.81**	0.7225 (0.6810, 0.7946)	0.2494
**Ours**	**76.43**	71.41	**80.36**	74.16	**81.74**	0.7422 (0.6878, 0.7981)	**-**

Values in bold black font represent the best performance in each column.

## Discussion

The presence of MVI is a significant predictor of postoperative recurrence in HCC patients, and its identification before surgery can improve personalized surgical planning, ultimately enhancing patient survival. While radiomics and deep learning have been used to predict MVI in HCC, they have limitations. Radiomics relies on laboriously hand-crafted feature extractors, leading to redundant extracted features. Meanwhile, deep learning with single slice or volume data inputted lacks information on the entire lesion or is computationally expensive. To address these challenges, we developed a dual-stream multiple instance CNN with modality attention and achieved good performance.

The MRI was utilized to predict MVI in HCC due to its ability in multimodal and multidirectional evaluation of lesions, providing more precise insights into soft tissue characterization, atomic signal intensity, lesion enhancement, and tissue function. Moreover, five commonly used MR sequences including T2-weighted, arterial phase, venous phase, delay phase, and apparent diffusion coefficient images were incorporated. The superior results were achieved by combining these modalities compared to using a single modality alone. To further enhance our approach’s clinical relevance, an attention mechanism was employed to prioritize the crucial modality. The performance of various MRI modalities were assessed and the results were consistent with the doctors’ experience that the arterial phase sequence exhibited better performance compared to the delay phase, venous phase, ADC, and T2 sequence. A possible explanation is that the arterial phase of contrast-enhanced MRI displays changes in MVI and surrounding tissue enhancement features (28), which can be captured by our model. However, the quality of the arterial phase image can be affected by many factors, such as the doctor’s manipulation, the patient’s blood flow status, and the amount and rate of contrast agent injection. Multi-arterial phase image can not only avoid the above problems, but also obtain abundant blood flow information (29). Therefore, using multi-arterial phase image for microvascular prediction might obtain better prediction results.

In MIL, instance-wise max-pooling (30) and average pooling (31) are two widely used methods for aggregating information from instances within a bag. Specifically, max-pooling only retains the instance embedding with the highest score, while average pooling treats each instance as equally important. However, neither of these methods considers the relationships between instances during MIL inference (32). The dual-stream aggregator in this study modeled the instance-to-instance relationships by calculating the similarity between two queries using a trainable distance measurement. As a result, instances that are more similar to the critical instance were given greater consideration.

The performance of a model can be greatly affected by the choice of feature extractor, especially when working with small medical image datasets. Some studies (23, 33) have used pre-trained backbones on natural images as feature extractors, but this approach may not always yielding optimal results. In our study, we attempted to use a pre-trained ResNet as a feature extractor, but found unsatisfactory results, potentially due to overfitting caused by the network’s depth. Instead, we found that a 5-layer CNN designed as a feature extractor performed better. This finding is consistent with research (34) which demonstrated that a shallow CNN trained from scratch can outperform a pre-trained deep model. By customizing the feature extractor to the specific task, we achieved superior results in predicting MVI for HCC patients.

Compared to radiomics, deep learning methods usually do not require precise lesion annotation, saving time and labor. In our study, we utilized a square bounding box that fit the entire tumor and covered the ROI during image preprocessing. Previous studies (35) have suggested that peritumoral features may indicate the presence of MVI, but the effect of the margin used to crop the region is not yet confirmed. A small margin may sacrifice important MVI-related information, while a large margin may introduce redundant information that could affect model performance. In Liu’s research (14), margin was defined as the scale of the edge length of the labeled bounding box, and experiments were conducted with marginal values ranging from 0.6 to 1.0. The best performance was achieved with a margin of 0.8. Although we did not extensively study the impact of margin, we observed that utilizing a bounding box that is approximately 10mm larger than each tumor boundary significantly improved the outcomes compared to a uniform size (based on the maximum tumor size) for locating areas of interest across tumors.

In the comparison with other methods, we did not directly compare the results due to the use of different datasets and the incorporation of both medical image and clinical factors in some studies. Instead, to ensure fairness, we replicated all methods on our dataset and compared their performance. Furthermore, all methods were designed with five branches for feature extraction from five MRI modalities, which were then combined for MVI prediction. Liu et al. (14) only used a single slice of the lesion as input, ignoring contextual information about the lesion. While Zhang et al. (16), Jiang et al. (17), and Zhou et al. (15) inputted information about the entire or partial volume of the lesion, the 3D CNNs used were usually designed to be shallow due to high computational resource requirements and difficulty in training, which may result in insufficient feature extraction. **Table 6** showed that although there was no significant difference in AUC, our method overall performs better.

There are several limitations in our study that need to be addressed in future research. Firstly, the dataset used in our study was relatively small, which might have impacted the performance of our model. Therefore, it is necessary to obtain larger samples to improve the robustness of the model. Secondly, our model was trained and evaluated in a single-center setting with a single MRI scanner, which could potentially introduce data and model bias. To enhance the generality of the model, it is crucial to include more diverse and variable data from multiple centers and scanners. Thirdly, our study only focused on analyzing MRI images and did not consider important clinical information such as age, gender, AFP, Child-Pughscore, HBsAg, or HCsAg, which are potential indicators of MVI. Future studies should take these clinical factors into account to improve the accuracy and reliability of the model.

In conclusion, we developed a modality-based attention and dual-stream multiple instance CNN for predicting MVI of HCC. This method overcomes the limitations of previous studies with single slice or entire volume lesion inputted and exhibits promising performance, suggesting its potential as a supportive tool in clinical diagnosis.

## Data availability statement

The raw data supporting the conclusions of this article will be made available by the authors, without undue reservation.

## Ethics statement

Written informed consent was obtained from the individual(s) for the publication of any potentially identifiable images or data included in this article.

## Author contributions

Conception and design: JZ, JXi, ZL. Collection and assembly of data: JXu, JW and YW. Verification of the underlying data: JZ, JXu, ZL and YW. Development of methodology: JZ, JXi and ZL. Data analysis and interpretation: JZ, JXi and ZL. Writing original draft: JZ, JXi, ZL. All authors contributed to the article and approved the submitted version.
